# 

**Published:** 1860-10

**Authors:** 


### CONTENTS

OF THE

### icago Medical Journal for October, 1860.

### ORIGINAL COMMUNICATIONS.

TICLE 1.—Notes Relating to the Extirpation of the Parotid Gland. By Daniel Brainard, M. D., Prosessor of Surgery in Rush Medical College.................. 569
RTICLE 2.—Sympathetic Ophthalmitis, and Certain Forms of Chronic Iritis. By E. L. Holmes, M. D.......................................... 581
RTICLE 3.—Nervous Agency in Inflammatory Action. By L. S. Ellis, A. M., M. D......... 585
TICLE 4.—Diphtheria. By Dr. A. K. Van Horn.................................. 596

### SELECTED.

ality of Toads.................................................... 601
ificial Production of Bone by Transplantation of the Periosteum ..................... 602
erimental Pathology ................................................ 611
w Method of Dissection ............................................... 614
oduction of the Tea Plant into the United States ............................. 616
h by Chloroform .................................................. 618

### EDITORIAL.

ook and Pamphlet Notices........................................ 619 to 628

ew Needle for Sutures................................................ 629

eedings of the Galesburg Medical Society ..................................... 632

